# The Indirect Impact of COVID-19 on Major Clinical Outcomes of People With Parkinson's Disease or Parkinsonism: A Cohort Study

**DOI:** 10.3389/fneur.2022.873925

**Published:** 2022-05-16

**Authors:** Luca Vignatelli, Flavia Baccari, Laura Maria Beatrice Belotti, Corrado Zenesini, Elisa Baldin, Giovanna Calandra-Buonaura, Pietro Cortelli, Carlo Descovich, Giulia Giannini, Maria Guarino, Giuseppe Loddo, Stefania Alessandra Nassetti, Luisa Sambati, Cesa Scaglione, Susanna Trombetti, Roberto D'Alessandro, Francesco Nonino

**Affiliations:** ^1^UOSI Epidemiologia e Statistica, IRCCS Istituto delle Scienze Neurologiche di Bologna, Bologna, Italy; ^2^Dipartimento di Scienze Biomediche e NeuroMotorie, Università degli Studi di Bologna, Bologna, Italy; ^3^AUSL di Bologna, Bologna, Italy

**Keywords:** COVID-19, Parkinson's disease, parkinsonism, cohort studies, physiotherapy

## Abstract

**Background:**

The indirect impact of the COVID-19 epidemic on major clinical outcomes of people with Parkinson's disease (PD) or other parkinsonism is unknown.

**Objectives:**

The study aimed to (1) describe changes in healthcare services during the first epidemic bout in people with PD or parkinsonism; (2) compare the occurrence of hospitalization for any PD-related major clinical outcomes in 2020 with 2019; (3) investigate the factors, including changes in healthcare services, associated with major clinical outcomes and death.

**Methods:**

All healthcare services of the province of Bologna and major clinical outcomes were assessed through a record linkage study (ParkLink Bologna) using clinical data and health databases. Same analyses were performed in a random cohort of controls matched for age, sex, district of residence, and comorbidities with the ParkLink cohort (ratio of 1:10).

**Results:**

A cohort of subjects with PD (759) or other parkinsonism (192) was included together with a cohort of controls (9,226). All indicators of healthcare services dropped at least below 50% during the lockdown period in all cohorts, mostly impacting physiotherapy in people with PD (−93%, 95% CI 88–96%). In 2020, compared to 2019, a three-fold risk of major injuries (RR 3.0, 95% CI 1.5–6.2) and infections (RR 3.3, 95% CI 1.5–7.2), excluding COVID-19, was observed only in people with PD, and neither in people with parkinsonism nor in controls. Decreased physiotherapy was associated with the occurrence of at least one major clinical outcome (OR 3.3, 95% CI 1.1–9.8) in people with PD. Experiencing at least one major clinical outcome was the strongest risk factor for death (OR 30.4, 95% CI 11.1–83.4) in people with PD.

**Conclusions:**

During the first COVID-19 epidemic peak, healthcare services were drastically reduced in a province of northern Italy, regardless of the disease condition. However, compared to 2019, in 2020, only people with PD had a higher risk of major clinical outcomes, that were associated with higher mortality. Strategies to maintain physical activity in people with PD should be implemented in possible future health emergencies.

## Introduction

The COVID-19 pandemic has been heavily striking the majority of countries globally since early 2020 (1). The European community of neurologists is putting an extraordinary effort to monitor the neurological symptoms and complications in patients with COVID-19 (2). However, besides the direct health burden due to the infection, restriction measures, prompted by the need for distancing, are possibly impacting many determinants of health and wellbeing (3), such as (1) social factors, (2) economic factors, (3) environmental factors, (4) individual health behaviors, and (5) access to health and social care services. The latter, in particular, was limited worldwide during the pandemic up to May 2020 for all kinds of conditions (4). Although it has been suggested that restrictions on healthcare services may be particularly burdensome for people with chronic disorders (3), the magnitude of the indirect impact of the pandemic in terms of major clinical outcomes is still not clear. Chronic neurodegenerative diseases may be a paradigmatic example of increased vulnerability during the abrupt rearrangement of healthcare services due to emergencies such as a pandemic bout (5).

Parkinson's disease (PD), atypical parkinsonism (AP), and vascular parkinsonism (VP) are neurologic chronic disorders affecting mainly older persons burdened by progressive disability, comorbidities, higher polypharmacy risk, and requiring follow-up and supportive care provided in specialized healthcare settings (6). Governmental control measures, limiting social interactions and mobility, and the restriction of healthcare services through the cancellation of outpatient appointments and scheduled hospital admissions could have caused both a reduction of individual healthy behavior (e.g., regular physical activity) as well as a limitation of timely neurologic counseling and physiotherapy.

During the first outbreak peak, from March 1 to May 31, 2020, Italy was the most hit country in Europe. The city of Bologna, the regional capital city of the Emilia-Romagna region (north-east of Italy), counted 4,636 cases in the same period. Starting from March 9, 2020, national governmental authorities imposed strict control measures limiting social interactions and mobility for the whole Italian population. Leaving home was banned except for buying food or drugs or for health reasons and only community services workers (food chain and health, energy, communication, or security services) were allowed to circulate. All routine outpatient healthcare activities were canceled or postponed by local health authorities to contain the number of outpatients accessing healthcare facilities, hoping to reduce the spreading of infection. In particular, the Local Health Trust of Bologna (LHTB) recommended all health facilities cancel non-urgent scheduled visits, tests, and admissions, and reduce the availability of high-priority visits and tests.

We hypothesize that strict lockdown measures and healthcare organizational changes due to the COVID-19 epidemic may have worsened major clinical outcomes among patients with PD, AP, or VP. Thus, we designed a study with the following aims: (1) to describe healthcare services change in the first epidemic bout, including the lockdown period and the next period with lower epidemic risk, in cohorts of people with PD, AP, or VP [ParkLink Bologna cohort (7)] and a matched general population control cohort; (2) to compare the occurrence of PD-related major clinical outcomes of the first 7 months of the epidemic in 2020 with the same period of 2019, in the same cohorts; (3) to analyze the demographic and clinical factors associated with PD-related major clinical outcomes and death in the PD cohort.

## Materials and Methods

The STROBE (Strengthening the Reporting of Observational Studies in Epidemiology) (8) and the RECORD (The REporting of studies Conducted using Observational Routinely-collected health Data) (9) guidelines were followed.

### Study Design

**Figure 1:**
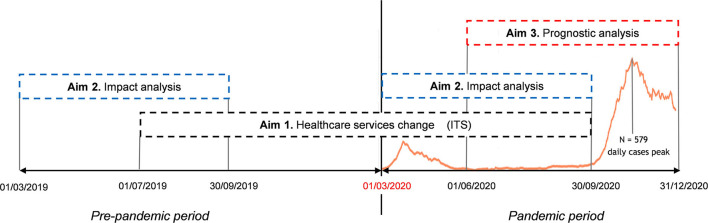
The study analysis plan of the study is according to the three aims. Aim 1—Healthcare services change: interrupted time-series (ITS) design applied to the four cohorts (PD, AP, VP, and controls) from July 1, 2019, to September 30, 2020. Aim 2—Impact analysis: historical cohort design applied to the three cohorts comparing the period March to September 2020 with the same period of 2019. Aim 3—Prognostic analysis: historical cohort design applied to the PD cohort from June 1, 2020, to December 31, 2020. The absolute number of SARS-CoV-2 infected people is reported in orange color in the time scale.

Healthcare services change (aim 1): Interrupted time-series (ITS) design was applied to four cohorts (PD, AP, VP, and controls) from July 1, 2019 to September 30, 2020, excluding the second epidemic bout (approximately from October 2020 on) to avoid confounding (Figure 1). A pre-epidemic period (spanning from July 1, 2019 to February 29, 2020) and an epidemic period (from March 1, 2020 to September 30, 2020) were considered. The latter included the first epidemic bout (from March 1, 2020 to May 31, 2020) and two phases of healthcare services rearrangement: a restriction phase (from March 9, 2020 to July 31, 2020) and a recovery phase (from August 1, 2020 to September 30, 2020). The index week change was set for March 9–15, 2020, corresponding to the first week of lockdown.

Impact analysis (aim 2): Historical (retrospective) cohort design was applied to the four cohorts (Figure 1) comparing the period from March 1, 2020 to September 30, 2020, with the same period of 2019.

Prognostic analysis (aim 3): Historical (retrospective) cohort design was applied to the PD cohort (Figure 1) from June 1, 2020, to December 31, 2020.

### Setting and Study Population

The local health trust of Bologna (LHTB), Northern Italy, had an adult population of 752,104 on December 31, 2019. This study is based on the ParkLink Bologna project (10), an ongoing record linkage system started in 2015, including consecutive prevalent and incident cases of PD or parkinsonism after signing a consent form, living in the LHTB area. For this study, we included people with PD or parkinsonism and people anonymously matched from the general population (control cohort), alive on March 1, 2020. Thirty neurologists operating in the LHTB area, including three hospital-based movement disorders outpatient services and several public and private outpatient services, voluntarily joined the project. Diagnosis of PD or parkinsonism is defined prospectively by the recruiting neurologist during the day-by-day clinical practice. Neurologists were requested to apply Gelb criteria (11) for PD and other international criteria for pre-specified types of parkinsonism [multiple system atrophy (12), progressive supranuclear palsy (13), and for VP (14)]. The first two and other rare or unspecified neurological causes affecting basal ganglia are labeled for this study as parkinsonism. Educational activities on the application of more recent international diagnostic criteria are ongoing (15, 16). Drug-induced parkinsonism is excluded. The following data have been recorded in an electronic case report form, linked to administrative databases, and stored in a secure database: unique anonymous identification code, date of birth, diagnosis, year of onset, motor symptoms at onset (tremor/bradykinesia), side of onset (unilateral/bilateral), and Hoehn and Yahr score. For all clinical data, the coverage is 100% but motor symptoms at onset (tremor/bradykinesia) have 14% of missing data.

The control cohort included a random sample of people matched with the ParkLink cohort, with a ratio of 1:10 for age, sex, district of residence, and comorbidity according to the Charlson Index (17). Subjects that used drugs for PD (levodopa, dopamine agonists, and monoamine oxidase-B inhibitors) for at least 180 consecutive days during 2019 were considered to be affected by PD or AP and excluded.

### Data Sources

As the Italian health system is universal, all accesses to any public or private health facility are recorded in a homogenous way, and personal data are stored by the qualified local health trust in secure databases. The Unità Operativa Epidemiologia e Statistica of the IRCCS Istituto delle Scienze Neurologiche di Bologna (part of the LHTB) is in charge of the ParkLink Bologna project and has access to several health administrative databases (outpatient tests and visits, ED admissions, hospital discharge, drug prescription, and mortality). The health data used by this study have almost 100% coverage as recording is mandatory at any access. The ParkLink Bologna project allows, for each individual, the linkage of the clinical diagnosis with administrative databases and the extraction of population-matched controls.

### Healthcare Services Change Measures (Aim 1)

The following were considered as measures of healthcare services: any outpatient visit, any neurologic outpatient visit, any outpatient physical therapy visit or activity, any test (lab/diagnostic/neuroradiologic), any non-urgent hospital admission, overall prescription of any drug, and drugs specific for PD, calculated as days of therapy based on Defined Daily Doses (see Table 1).

**Table 1:** Outcome measures and corresponding codes of identification for each aim.

**Aim**	**Outcome measure**	**Codes**
1. Healthcare services change	Any outpatient visit	Any visit recorded in the outpatient specialist services database
	Any neurologic outpatient visit	Any neurologic visit recorded in the outpatient specialist services database
	Any outpatient physical therapy evaluation or treatment	Any physical therapy visit or activity recorded in the outpatient specialist services database
	Any test	Any test recorded in the outpatient specialist services database
	Any non-urgent hospital admission	Any scheduled admission recorded in the hospital discharge administrative database
	Any drug prescription^a^	Any prescription recorded in the drug prescription databases
	Parkinson's disease drug prescription^a^	ATC codes: N04BA02, N04BA03 and N04BA05 for Levodopa; N04BC04, N04BC05, N04BC01 and N04BC09 for dopamine agonists; N04BD01, N04BD02 and N04BD03 for MAO inhibitors
2. Impact analysis	Any urgent hospital admission	Any urgent admission recorded in the hospital discharge administrative database
	Hospital admissions for major injuries	ICD-9-CM codes: 800–829 (fracture of skull/of spine and trunk/of upper and lower limb); 850–854 (intracranial injury excluding those with skull fracture); 717 (internal derangement of knee); 920 (contusion of face, scalp, and neck except eye(s)); 905.2–905.4 (late effect of fracture of neck of femur/of lower extremities); 733.13 (pathologic fracture of vertebrae); 922.31 (contusion of back); 924.01 (contusion of hip); 959.11–959.12 (other injury of chest wall/of abdomen); 959.19 (other injury of other sites of trunk)
	Hospital admissions for infections^b^	ICD-9-CM codes: 480–486, 466, 507 (pneumonia); 038 (septicaemia); 595 (cystitis); 707 (decubitus)
	Hospital admissions for gastro-intestinal events	ICD-9-CM codes: 560 (intestinal obstruction); 562 (diverticular disease); 567 (peritonitis); 569 (intestinal or peritoneal abscess/intestinal perforation/intestinal fistula); 578 (diverticular bleeding)
	Hospital admissions for thromboembolic events	ICD-9-CM codes: 451 (deep vein thrombosis); 415 (pulmonary embolism)
	Hospital admissions for psychiatric events	ICD-9-CM codes: 290–299, 300–315.9 (psychosis)
	Hospital admissions for hypotension/syncope	ICD-9-CM codes: 780.2 (hypotension); 458 (syncope)
	Hospital admissions for acute cardiac and cerebrovascular events	ICD-9-CM codes: 410–411, 428 (acute cardiac events); 430–434, 436 (acute cerebrovascular events).
3. Prognostic analysis	All categories related to aim 2 were merged as composite any PD related major clinical outcome	Same as above

a *Calculated as days of therapy on the basis of Defined Daily Doses*.

b *Discharge codes related to COVID-19 events were excluded (079.82; 480.3)*.

### Outcome Measures for Impact Analysis (Aim 2)

Infections, injuries, cardiovascular events, neuropsychiatric, and gastrointestinal events are among the most common reasons for hospital admission (18) in people with PD. Thus, the following major clinical outcomes were identified according to ICD-9-CM codes (see Table 1): any urgent hospital admission; hospital admissions for major injuries, infections, gastro-intestinal events (intestinal obstruction with complications), thromboembolic events (deep vein thrombosis; pulmonary embolism), psychiatric events, hypotension/syncope, acute cardiac events, and acute cerebrovascular events. The earlier reported reasons for hospital admissions were merged as a composite measure (any PD-related major clinical outcome). Discharge codes related to COVID-19 events were excluded.

### Outcome Measures for the Prognostic Analysis (Aim 3)

The earlier reported composite measure (any PD-related major clinical outcome) and death for any reason.

### Statistical Analysis

The synopsis of the statistical plan, put in the context of the timeline of the epidemic, is reported in Figure 1.

To evaluate *healthcare services change* (aim 1), ITS design, based on weekly (Monday to Sunday) events from July 1, 2019 to September 30, 2020, was performed. The dependent variables (healthcare services) were measured before and during the lockdown (event considered as “intervention”), splitting the time series. The pre-intervention period was from July 1, 2019 to March 8, 2020; the intervention (index week change) was set in the week of March 9–15, 2020; the post-intervention period was from March 16, 2020 to September 30, 2020. The generalized linear regression model was fitted to the weekly Poisson counts (19). Person-time at-risk (log-transformed) was used as an offset variable. A scaling adjustment was made to correct the model for overdispersion. Autocorrelation was detected by inspecting autocorrelation graphs of residuals; adjustment of seasonality was made using the Fourier terms function. The level of change after the index week and the weekly change in the post-interruption slope is reported as Incidence Rate Ratio (IRR), with 95% confidence intervals (95% CI).

In the *impact analysis* (aim 2), the outcome measures (see above) recorded between March 1, 2020 and September 30, 2020, were compared with those of 2019. Monthly rates were calculated on the average of the 7-month period, using as numerator the number of events recorded and as denominator the person-time at-risk in each month. Rate ratios point estimates and 95% CI, between periods (2020 vs. 2019) and among conditions (PD/AP/VP/controls), were used to test for differences in outcome measures.

In the *prognostic analysis* (aim 3), a *post-hoc* multivariable logistic model was performed only in the PD cohort to evaluate the variables associated with the occurrence of the composite “any PD related major clinical outcome,” during the period June 1, 2020 and September 30, 2020. The reduction of healthcare services was considered as an individual risk factor and calculated, by subject, as the difference between the number of supplies recorded during the first lockdown period (March 1–May 30, 2020) and the number of supplies in the corresponding period of 2019. This difference was then transformed into three categories: “increase,” when the number of supplies in 2020 was higher than those in 2019; “reduction,” when the number of supplies in 2020 was lower than those in 2019; “no change” (reference category) when the number of supplies was the same in the two periods. The multivariable model was adjusted for the number of supplies between March 1, 2019 and May 30, 2019 (baseline), and for the level of disability, retrieved from the national social security dataset and dichotomized as < or ≥67% according to the quantitative parameters defined by the Italian social security system.

A *post-hoc* multivariable logistic model was performed in the PD cohort to analyze the variables associated with death for any reason between June 1, 2020 and December 31, 2020.

Data linkage and statistical analyses were conducted using Stata SE version 14.2.

## Results

On March 1, 2020, the ParkLink Bologna record linkage system counted 1,255 participants. After excluding those dead before the start of the study (218) and those pending diagnostic definition (86), the ParkLink cohort included 759 subjects with PD (mean age 75.3 years), 93 with AP (78.5 years), and 99 with VP (83.2 years). The control cohort included 9,226 subjects (76.3 years). Demographic and clinical characteristics are summarized in Table 2. People with AP and VP were older and had more comorbidities (congestive heart failure, cerebrovascular disease, dementia, chronic pulmonary disease, diabetes, and renal disease). Compared to PD, they had a higher mean age at onset, different clinical features, and different drug treatment patterns.

**Table 2:** Demographic and clinical features of the ParkLink cohorts and the control cohort.

**Demographics**	**Control** **cohort**	**Parkinson's disease cohort**	**Atypical parkinsonism cohort**	**Vascular** **parkinsonism** **cohort**	***P***
*N*	9,226	759	93	99	
Mean age, yrs (SD, range)	76.3 (9.4, 40–98)	75.3 (9.5, 40–96)	78.5 (7, 45–85)	83.2 (6, 66.1–97.5)	<0.001
Mean age at onset, yrs (SD, range)		66.3 (10.5, 29–89)	70.6 (7.3, 45–85)	75.6 (6.7, 53–89)	<0.001
**Age distribution**, ***n*** **(%)**					<0.001
40–49 yrs	137 (1.5)	13 (1.7)	0 (0.0)	0 (0.0)	
50–59 yrs	406 (4.4)	39 (5.1)	1 (1.1)	0 (0.0)	
60–69 yrs	1,420 (15.4)	136 (17.9)	11 (11.8)	2 (2.0)	
70–79 yrs	3,369 (36.5)	283 (37.3)	36 (38.7)	22 (22.2)	
80–89 yrs	3,504 (38.0)	267 (35.2)	42 (45.2)	59 (59.6)	
≥90 yrs	390 (4.2)	21 (2.8)	3 (3.2)	16 (16.2)	
**Sex**, ***n*** **(%)**					0.363
Male	5,344 (57.9)	446 (58.8)	47 (50.5)	62 (62.6)	
Female	3,882 (42.1)	313 (41.2)	46 (49.5)	37 (37.4)	
**District**, ***n*** **(%)**					0.406
Bologna	4,129 (44.8)	335 (44.1)	50 (53.8)	37 (37.4)	
Reno	1,098 (11.9)	94 (12.4)	7 (7.5)	13 (13.1)	
Pianura Est	1,658 (18.0)	131 (17.3)	15 (16.1)	27 (27.3)	
Pianura Ovest	1,023 (11.0)	86 (11.3)	9 (9.7)	12 (12.1)	
Appennino	642 (7.0)	59 (7.8)	4 (4.3)	2 (2.0)	
San Lazzaro	676 (7.3)	54 (7.1)	8 (8.6)	8 (8.1)	
**Charlson index**, ***n*** **(%)**					<0.001
0	7,312 (79.3)	619 (81.6)	59 (63.4)	66 (66.7)	
1	930 (10.1)	66 (8.7)	18 (19.3)	12 (12.1)	
2	697 (7.6)	51 (6.7)	10 (10.8)	12 (12.1)	
≥3	287 (3.0)	23 (3.0)	6 (6.5)	9 (9.1)	
**Comorbidities**, ***n*** **(%)**					
Myocardial infarction	178 (1.9)	12 (1.6)	1 (1.1)	2 (2.0)	0.872
Congestive heart failure	453 (4.9)	40 (5.3)	10 (10.8)	10 (10.1)	0.011
Peripheral vascular disease	127 (1.4)	5 (0.7)	1 (1.1)	0 (0.0)	0.279
Cerebrovascular disease	392 (4.3)	18 (2.4)	10 (10.8)	11 (11.1)	<0.001
Dementia	158 (1.7)	30 (4.0)	11 (11.8)	11 (11.1)	<0.001
Chronic pulmonary disease	264 (2.9)	11 (1.5)	4 (4.3)	6 (6.1)	0.012
Peptic ulcer disease	27 (0.3)	3 (0.4)	1 (1.1)	1 (1.0)	0.129
Liver disease	33 (0.4)	1 (0.1)	0 (0.0)	0 (0.0)	0.746
Diabetes	320 (3.5)	22 (2.9)	8 (8.6)	9 (9.1)	0.002
Renal disease	141 (1.5)	14 (1.8)	0 (0.0)	5 (5.1)	0.044
Any malignancy	500 (5.4)	37 (4.9)	5 (5.4)	3 (3.0)	0.758
**Clinical features at onset**, ***n*** **(%)**					
Unilateral		625 (82.3)	24 (25.8)	47 (47.5)	<0.001
Bilateral		134 (17.7)	69 (74.2)	52 (52.5)	
Tremor (yes)		481 (72.0)	32 (45.7)	47 (58.8)	<0.001
Bradykinesia (yes)		521 (80.2)	80 (94.1)	72 (82.8)	0.003
Mean Hohen-Yahr score, (SD, range)		2.5 (1, 1–5)	3.5 (1.2, 1–5)	2.8 (1, 1–5)	<0.001
**Etiology in atypical parkinsonism**, ***n*** **(%)**					
Progressive supranuclear palsy			16 (17.2)		
Multiple system atrophy			10 (10.8)		
Other/undetermined			67 (72.0)		
**Drug treatment**, ***n*** **(%)**					<0.001
No therapy		71 (9.4)	23 (24.7)	20 (20.2)	
Levodopa only		371 (48.8)	42 (45.2)	59 (59.6)	
Dopaminergic only or IMAO B only		28 (3.7)	4 (4.3)	1 (1)	
Any combination of drugs		289 (38.1)	24 (25.8)	19 (19.2)	

### Healthcare Services Change: Interrupted Time Series Before and After the Epidemic Bout

**Figure 2:**
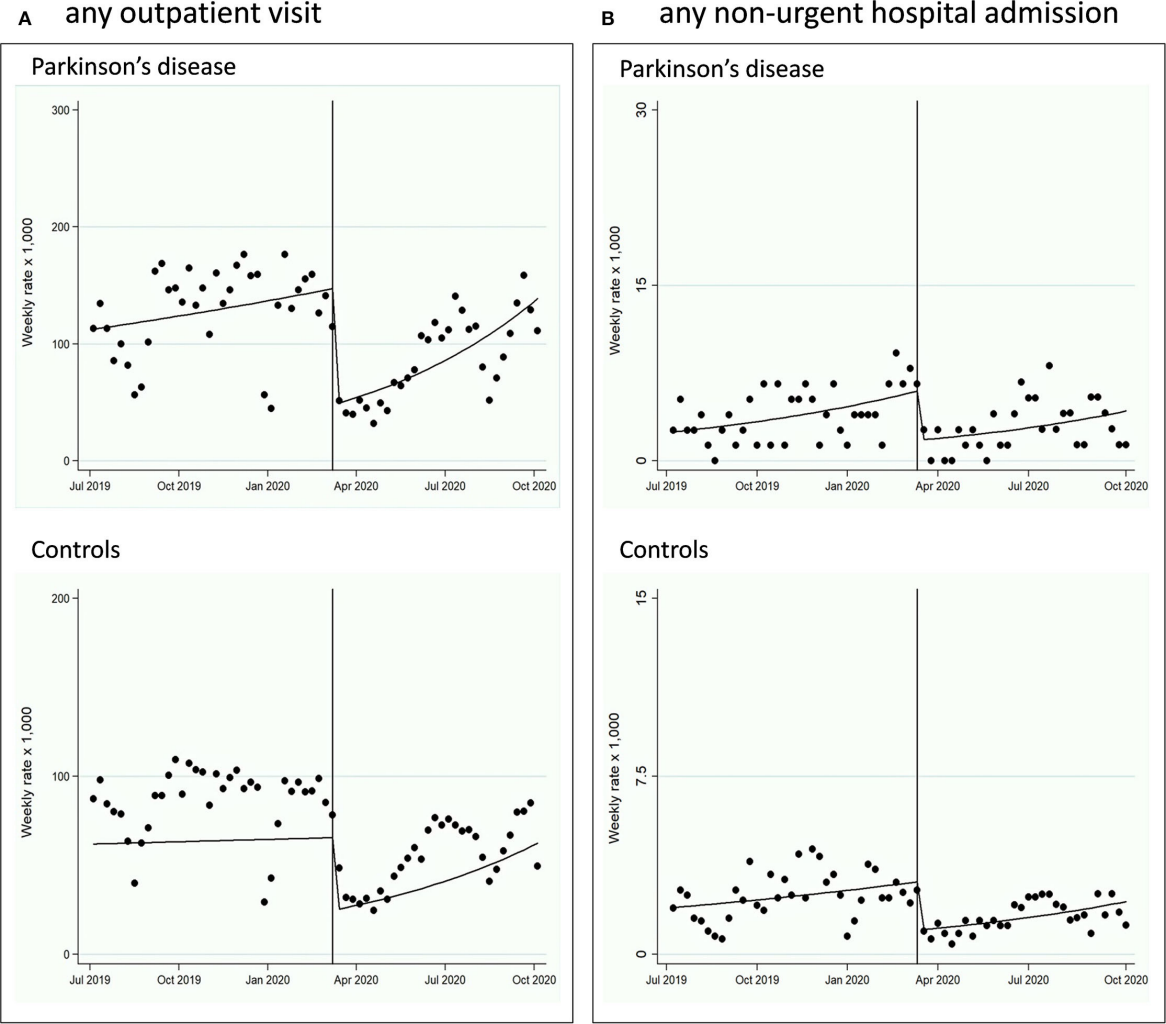
Interrupted time-series (ITS) design, from July 1, 2019, to September 30, 2020, evaluating the healthcare services change in the PD and control cohorts. The pre-intervention period was from July 1, 2019, to March 8, 2020; the intervention (index week change) was set in the week of March 9 to 15, 2020; the post-intervention period was from March 16, 2020, to September 30, 2020. **(A)** Any outpatient visit change is reported. **(B)** Any non-urgent hospital admission change is reported.

**Figure 3:**
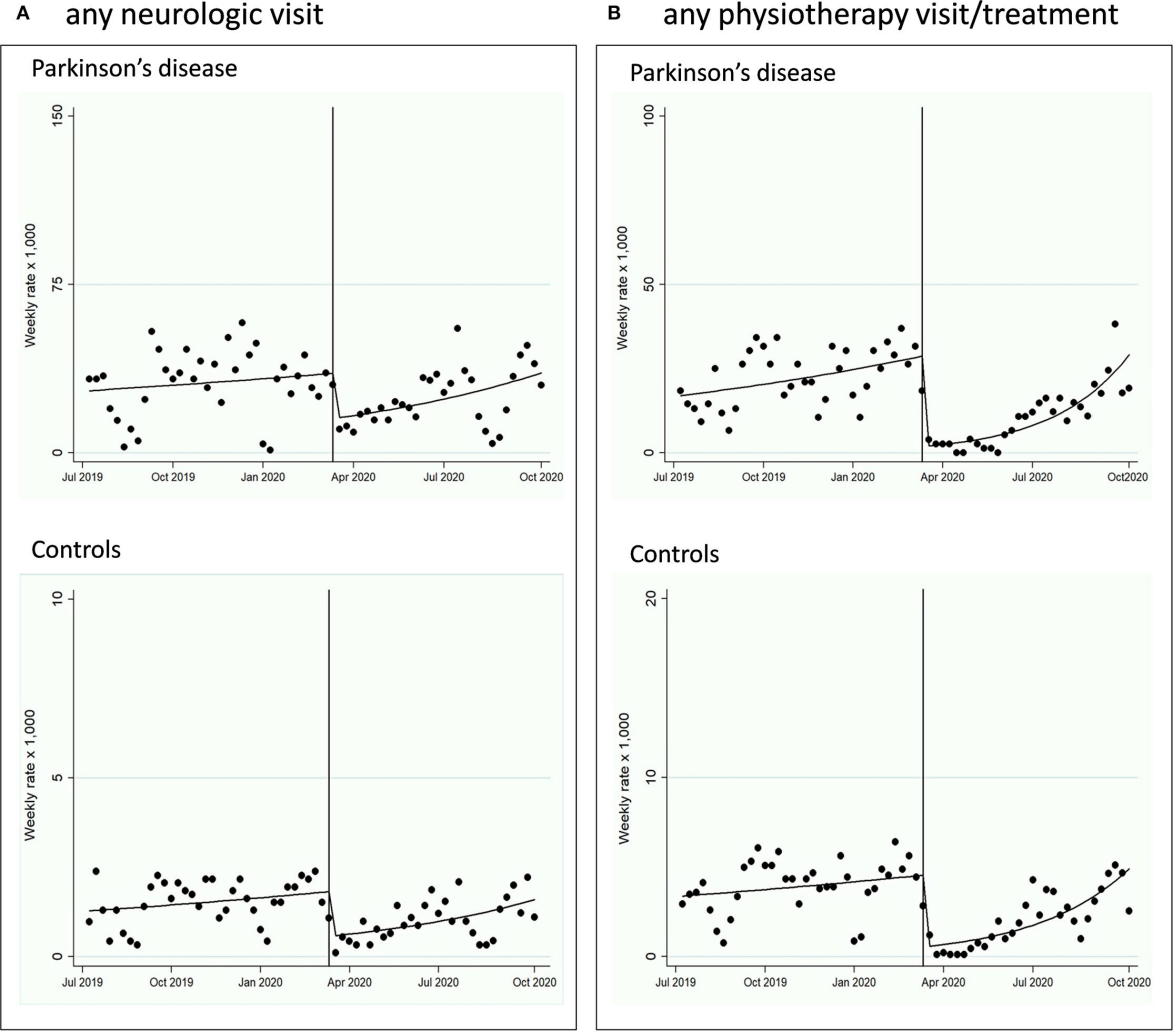
Interrupted time-series (ITS) design, from July 1, 2019, to September 30, 2020, evaluating the healthcare services change in the PD and control cohorts. The pre-intervention period was from July 1, 2019, to March 8, 2020; the intervention (index week change) was set in the week of March 9–15, 2020; the post-intervention period was from March 16, 2020, to September 30, 2020. **(A)** Outpatient neurologic visit change is reported. **(B)** Outpatient physiotherapy evaluation or treatment change is reported.

In the PD, AP, and VP cohorts (Figures 2, 3; Supplementary Material; Table 3), all indicators of healthcare services (any outpatient visit, outpatient neurologic visit, outpatient physiotherapy evaluation or treatment, any outpatient testing, any non-urgent hospital admission) dropped drastically after the index week change (March 9–15, 2020) until September 30, 2020, with IRR ranging from 0.07 (95% CI 0.04–0.12) for any outpatient physiotherapy evaluation or treatment in PD to 0.54 (0.34–0.86) for any outpatient exam in VP. The trend after the index week change slightly increased for almost all outpatient healthcare service measures (2–8% increase per week), but not for non-urgent hospital admissions. The control cohort showed similar reduction and trends (Figures 1, 2; Table 3). The mean days of prescription of any drugs did not change in PD (433.3, SD 270.4, in 2019, vs. 451.7, SD 359.3, in 2020, *p* = 0.27), AP (535.6, SD 301.4, in 2019, vs. 478.1, SD 362.5, in 2020, *p* = 0.16), and VP (579.3, SD 280.6, in 2019, vs. 609.4, SD 343.7, in 2020, *p* = 0.83), and slightly increased in controls (423.2, SD 312.2, in 2019, vs. 435.5, SD 325.1, in 2020, *p* = 0.020). Prescription of drugs specific for PD did not change in PD (153.3, SD 123.6, in 2019, vs. 150.8, SD 117.1, in 2020, *p* = 0.704), AP (129.5, SD 102.0, in 2019, vs. 117.3, SD 95.6, in 2020, *p* = 0.54), and VP (75.8, SD 71.4, in 2019, vs. 72.0, SD 59.0, in 2020, *p* = 0.72).

**Table 3:** Interrupted time series of healthcare services measures in the ParkLink cohorts (Parkinson's disease, atypical parkinsonism, vascular parkinsonism) and the control cohort, local health trust of Bologna, from July 1, 2019 to September 30, 2020.

**Healthcare services measures**	**Parkinson's disease**		**Atypical parkinsonism**		**Vascular parkinsonism**		**Control**	
	**IRR**	**95% CI**	**IRR**	**95% CI**	**IRR**	**95% CI**	**IRR**	**95% CI**
**Any outpatient visit**								
Pre-post change	0.33	0.24–0.44	0.30	0.19–0.50	0.45	0.27–0.77	0.41	0.32–0.53
Weekly change in trend post-interruption	1.03	1.01–1.04	1.04	1.01–1.06	1.03	1.01–1.06	1.02	1.01–1.04
**Outpatient neurologic visit**								
Pre-post change	0.43	0.27–0.70	0.23	0.07–0.80	0.37	0.15–0.90	0.31	0.19–0.51
Weekly change in trend post-interruption	1.02	1.00–1.05	1.05	0.98–1.11	1.03	0.98–1.08	1.02	1.00–1.05
**Outpatient physiotherapy evaluation/treatment**								
Pre-post change	0.07	0.04–0.12	0.14	0.04–0.47	0.41	0.15–1.13	0.12	0.07–0.21
Weekly change in trend post-interruption	1.08	1.05–1.11	1.04	0.98–1.10	0.99	0.94–1.05	1.07	1.04–1.10
**Any outpatient exam**								
Pre-post change	0.40	0.30–0.54	0.53	0.31–0.89	0.54	0.34–0.86	0.38	0.30–0.47
Weekly change in trend post-interruption	1.03	1.01–1.04	1.02	0.99–1.05	1.02	0.99–1.04	1.03	1.02–1.04
**Any non-urgent hospital admission**								
Pre-post change	0.30	0.16–0.55	0.22	0.02–2.25	0.38	0.06–2.42	0.34	0.22–0.50
Weekly change in trend post-interruption	1.00	0.97–1.04	1.03	0.91–1.16	0.97	0.87–1.08	1.01	0.99–1.04

*Date change: week March 9–15, 2020; IRR, incidence rate ratio*.

### Impact Analysis: Major Clinical Outcome Rates Comparison Between 2019 and 2020 Periods

Any PD-related major clinical outcome significantly increased with a RR of 2.1 (95% CI 1.5–3.0) from March to September 2020 compared to the same period of 2019 (Table 4), only in the PD cohort. This result was due to the increase in major injuries (3.0, 1.5–6.2) and infections (3.3, 1.5–7.2). Major injuries' increase was mainly driven by both upper limb injuries (5.7, 1.3–53.2) and lower limb injuries (5.6, 1.6–29.7). Infection increase was due to pulmonary infections (4.0, 1.5–10.6) and sepsis (2.6, 0.5–13.4). Other measures showed a trend in increase (any urgent hospital admission and hospital admissions for heart failure). The AP and VP cohorts showed fairly similar but not significant trends for infections and the VP cohort for heart failure. In the control cohort, none of these measures changed between the two periods.

**Table 4:** Monthly rates (×1,000), as mean of March-September period in 2019 (control period) and 2020 (first epidemic bout period and flattening of the epidemic curve period), in the ParkLink Bologna cohorts and in the control cohort.

**Outcomes**	**PD**	**AP**	**VP**	**Control**	**PD**	**AP**	**VP**	**Control**	**PD**	**AP**	**VP**	**Control**
	**2019 monthly rate** **× 1,000**				**2020 monthly rate** **× 1,000**				**2020 vs. 2019 rate ratios (95% CI)**			
**Any urgent hospital admission**	13.4	23	17.3	10.7	17.6	33.5	9.3	11.6	1.3 (1–1.8)	1.4 (0.70–3.1)	0.54 (0.17–1.6)	1.1 (0.98–1.2)
**Any disease-specific hospital admission**	8.8	16.9	23.1	9.2	18.4	22.9	10.9	9.9	**2.1 (1.5–3.0)**	1.4 (0.56–3.3)	0.47 (0.16–1.2)	1.1 (0.96–1.2)
Major injuries	1.9	-	13	1.9	5.7	3.5	3.1	1.7	**3.0 (1.5–6.2)**	-	0.24 (0.03–1.2)	0.93 (0.72–1.2)
Infections	1.5	7.7	1.4	1.8	4.9	12.3	4.7	2.3	**3.3 (1.5–7.2)**	1.6 (0.4–6.4)	3.2 (0.3–169.5)	1.3 (0.99–1.6)
Gastro-intestinal events	1.1	1.5	4.3	0.49	0.39	-	-	0.73	0.35 (0.07–1.7)	-	-	1.5 (0.94-2.3)
Psychiatric events	2.3	7.7	4.3	1.4	3.1	1.8	3.1	1.2	1.4 (0.66–2.9)	0.23 (0.05–2.1)	0.72 (0.06–6.3)	0.91 (0.67–1.2)
Hypotension	0.56	-	-	0.48	0.98	5.3	-	0.33	1.7 (0.41–7.3)	-	-	0.69 (0.40–1.2)
Thromboembolic events	-	-	-	0.12	0.20	-	-	0.21	-	-	-	1.7 (0.69-4.0)
Coronary artery disease	0.56	-	1.4	0.56	0.59	1.8	1.6	0.51	1.0 (0.21–5.2)	-	1.08 (0.01–84.5)	0.91 (0.56–1.5)
Heart failure	0.56	1.5	1.4	1.5	1.8	1.8	6.2	1.8	3.1 (0.85–11.5)	1.15 (0.01–30.1)	4.3 (0.43–212)	1.2 (0.90–1.6)
Ischaemic stroke	0.19	-	-	0.81	0.59	-	-	0.81	3.1 (0.32–30.0)	-	-	1.0 (0.68–1.5)
Haemorrhagic stroke	0.19	-	-	0.22	0.20	-	-	0.27	1.0 (0.07–16.6)	-	-	1.2 (0.61–2.5)

*The last three columns show rate ratios comparing 2020 monthly rates to 2019 monthly rates within each cohort. In bold statistically significant rate ratios*.

*PD, Parkinson's disease; AP, Atypical parkinsonism; VP, Vascular parkinsonism*.

### Prognostic Analysis in the Parkinson's Disease Cohort

In the period June to September 2020, 38 (5.1%) out of 747 PD patients, alive on June 1, 2020, experienced at least one of the major clinical outcomes: infection (15 subjects, 39.5%), major injury (11, 29.0%), heart failure (4, 10.6%), psychosis (3, 7.9%), stroke (2, 5.2%), hypotension/syncope (1, 2.6%), thromboembolism (1, 2.6%), and myocardial infarction (1, 2.6%). This group of patients had a more severe clinical status (Hoehn and Yahr scale score of 2.9 vs. 2.5, *p* = 0.022), more frequent bradykinesia at onset (100 vs. 79.5%, *p* = 0.002), disability (44.7 vs. 22.4%, *p* < 0.001), chronic pulmonary disease (7.9 vs. 1.0%, *p* < 0.011), and a larger proportion of subjects with reduction of number of physiotherapy evaluation or treatment in 2020 vs. 2019 (23.7 vs. 11.1%, *p* = 0.051), compared to patients not experiencing a major clinical outcome (see Table 5 for details). The multivariable logistic model (adjusted for age, Hoehn and Yahr scale score, disability, number of physiotherapy evaluations or treatments in 2019, and chronic pulmonary disease, see Table 6) showed an independent association between any PD-related major clinical outcome and reduction of the number of physiotherapy evaluations or treatments in 2020 (3.3, 1.1–9.8).

**Table 5:** Prognostic analysis in Parkinson's disease cohort, period June-September 2020.

	**Parkinson's disease developing disease-specific outcomes**	**Parkinson's disease not developing disease-specific outcomes**	***P***
*N*	38	709	
Mean Age, yrs (SD, range)	76.8 (8.0, 59–88)	75.1 (9.6, 40–96)	0.283
Mean Age at onset, yrs (SD, range)	67.1 (8.2, 48–81)	66.2 (10.6, 29–89)	0.607
**Age distribution**, ***n*** **(%)**			0.395
40–49 yrs	0 (0.0)	13 (1.8)	
50–59 yrs	1 (2.6)	38 (5.4)	
60–69 yrs	6 (15.8)	130 (18.3)	
70–79 yrs	11 (29.0)	268 (37.8)	
80–89 yrs	20 (52.6)	240 (33.9)	
≥90 yrs	0 (0.0)	20 (2.8)	
**Sex**, ***n*** **(%)**			0.215
Male	26 (68.4)	413 (58.3)	
Female	12 (31.6)	296 (41.7)	
**District**, ***n*** **(%)**			0.512
Bologna	19 (50.0)	312 (44.0)	
Reno	4 (10.6)	86 (12.1)	
Pianura Est	10 (26.3)	120 (16.9)	
Pianura Ovest	3 (7.9)	81 (11.4)	
Appennino	1 (2.6)	57 (8.1)	
San Lazzaro	1 (2.6)	53 (7.5)	
**Disability**			0.002
Yes	17 (44.7)	159 (22.4)	
**Charlson index**, ***n*** **(%)**			0.084
0	28 (73.6)	584 (82.4)	
1	2 (5.3)	61 (8.6)	
2	6 (15.8)	44 (6.2)	
≥3	2 (65.3)	20 (2.8)	
**Comorbidities**, ***n*** **(%)**			
Myocardial infarction	0 (0.0)	11 (1.6)	0.999
Congestive heart failure	2 (5.3)	35 (4.9)	0.712
Peripheral vascular disease	0 (0.0)	5 (0.7)	0.999
Cerebrovascular disease	1 (2.6)	17 (2.4)	0.614
Dementia	1 (2.6)	29 (4.1)	0.999
Chronic pulmonary disease	3 (7.9)	7 (1.0)	0.011
Peptic ulcer disease	1 (2.6)	2 (0.3)	0.145
Liver disease	0 (0.0)	1 (0.1)	0.999
Diabetes	1 (2.6)	20 (2.8)	0.999
Renal disease	2 (5.3)	12 (1.7)	0.156
Any malignancy	4 (10.5)	32 (4.5)	0.104
**Clinical features at onset**, ***n*** **(%)**			
Unilateral	34 (89.5)	580 (81.8)	0.281
Bilateral	4 (10.5)	129 (18.2)	
Tremor (yes)	27 (79.4)	446 (71.6)	0.323
Bradykinesia (yes)	30 (100.0)	484 (79.5)	0.002
**Hoehn and Yahr scale at latest observation**			
Mean (SD)	2.9 (0.9)	2.5 (1.0)	0.022
Score 4–5, *n* (%)	8 (21.1)	120 (16.9)	0.511
**Drug treatment**, ***n*** **(%)**			0.487
No therapy	3 (7.9)	68 (9.6)	
Levodopa only	15 (39.5)	349 (49.2)	
Dopaminergic only or IMAO B only	2 (5.3)	25 (3.5)	
Any combination of drugs	18 (47.3)	267 (37.7)	
**Change in the number of any outpatient visit (2020 vs. 2019)**, ***n*** **(%)**			0.563
No change	12 (31.6)	246 (34.7)	
Reduction	23 (60.5)	374 (52.8)	
Increase	3 (7.9)	89 (12.6)	
**Change in the number of neurologic outpatient visit (2020 vs. 2019)**, ***n*** **(%)**			0.999
No change	28 (73.7)	431 (60.8)	
Reduction	11 (29.0)	207 (29.2)	
Increase	4 (10.5)	71 (10.0)	
**Change in the number of physiotherapy visit/activity (2020 vs. 2019)**, ***n*** **(%)**			0.051
No change	28 (73.7)	611 (86.2)	
Reduction	9 (23.7)	79 (11.1)	
Increase	1 (2.6)	19 (2.7)	
**Change in the number of non-urgent hospital admission (2020 vs. 2019)**, ***n*** **(%)**			0.231
No change	35 (92.1)	666 (94.0)	
Reduction	1 (2.6)	30 (4.2)	
Increase	2 (5.3)	13 (1.8)	

**Table 6:** Multivariable logistic model of prognostic analysis on factors associated to the occurrence of any Parkinson's disease related major clinical outcome in the Parkinson's disease cohort, period June-September 2020.

	**Multivarible logistic model**			
	**OR**	**95% CI**		***P-*value**
Age, yrs	1.01	0.98	1.05	0.460
Disability, yes vs. no	2.14	1.01	4.53	0.046
Chronic pulmonary disease at Charlton Index, yes vs. no	7.90	1.82	34.21	0.006
Hoehn and Yahr scale at latest observation	1.14	0.81	1.62	0.457
Physiotherapy visit/activity in 2019	0.84	0.55	1.29	0.434
Change in the number of physiotherapy visit/activity (2020 vs. 2019)				
Reduction vs. no change	3.27	1.10	9.78	0.033
Increase vs. no change	1.01	0.13	8.09	0.988

*OR, odds ratio*.

Between June and December 2020, overall mortality was 3.7% (27 subjects out of 747): 44.4% in the group that experienced and 3.6% in the group that did not experience any major clinical outcomes (*p* < 0.001). The multivariable logistic model showed that the following parameters were associated with death: 1-year increase in age (OR 1.1, 95% CI 1.01–1.2), a 1-point increase in Hoehn and Yahr scale score (1.6, 1.02–2.3), cerebrovascular disorder history (10.0, 2.3–43.4), COVID-19 infection (8.3, 2.2–31.4), and any PD-related major clinical outcome (30.4, 11.1–83.4).

## Discussion

During the first 7 months of the COVID-19 epidemic bout, including 3 months of strict social lockdown measures and radical rearrangement of healthcare organization followed by 4 months of gradual recovery, we observed a marked reduction of all scheduled healthcare services in a cohort of PD or other parkinsonism of a large province of Northern Italy. In relative terms, change in service utilization was identical to that of a control cohort matched for sex, age, residence area, and comorbidity. Compared with the 2019 pre-pandemic period, during the epidemic bout, people with PD showed a higher risk of hospitalization for PD-related major clinical outcomes, namely a three-fold risk of major injuries and infections (other than COVID-19). Moreover, in people with PD, such outcomes were independently associated with a reduction in physical therapy evaluation or treatment from 2019 to 2020. Finally, a preceding major clinical outcome was the strongest predictor of death.

During the first epidemic bout (March-May 2020), with strict lockdown measures in many countries, a meta-analysis of worldwide studies (4) found a median reduction—compared to previous periods—of 37% of any healthcare service, highest for visits (42%) and lower for hospital admissions (28%), diagnostics (31%), and therapeutics (30%). In our cohorts, we found even greater reductions for visits (57–63%), admissions (66–70%), and diagnostics (47–62%) but not for drug prescriptions. Outpatient physical therapy was the most impacted service, particularly in the PD cohort (93% of reduction). Similar data on physiotherapy in people with PD were reported in France (20). Data from 105 countries and territories covering all WHO regions report that cross-sectoral services for neurological disorders were the most frequently disrupted services (62.9%), followed by emergency/acute care (47.1%) (21). Travel restrictions due to lockdowns (81.7%) and regulatory closure of services (65.4%) were the most commonly reported causes of disruption.

Few data are available about the indirect impact of the COVID-19 epidemic on non-communicable chronic disorders. Excess mortality from cardiovascular diseases (22, 23), diseases of the respiratory system (22), cancer (24, 25), and chronic liver disease (26), possibly due to the disruption of essential health services during pandemic peaks, was observed in some European countries and the USA. Isolation measures have damaged the cognitive and mental health of people with dementia (27).

People with PD are *per se* at higher risk of hospital admissions due to infections (18), falls and injuries (18, 28), gastrointestinal complications (18), neuropsychiatric problems (18), and hypotension (29), and possibly thromboembolic (30) or cardiovascular events (18). In the first 7 months of the epidemic, people with PD suffered a doubling of events compared with the same period of 2019. Namely, major injuries and infections tripled, driven by a five-fold increase in limb injuries and a four-fold increase in pulmonary infections (excluding COVID-19). A possible explanation is that forced immobility due to social restriction (especially during the first 3 months of the epidemic) and the almost complete cancellation of scheduled physical therapy may have impacted motor function and the ability to walk among patients with PD. This, in turn, could have increased the risk of falls and major injuries (especially limb fractures). Pneumonia, another marker of disease progression (18), could be a direct consequence of motor impairment after a major injury [e.g., a fracture (31)] and/or due to the reduction of primary care contacts (32). Some empirical data consistently show a reduction in the daily walking activity during the confinement period compared to the pre-confinement period (33), the reduction in the amount, duration, and frequency of exercise (34, 35) and physiotherapy (20) in people with PD, with possible subjective and objective worsening of both motor and non-motor features (20, 34, 36–39).

Physical exercise is one of the milestones of PD treatment (40), improving physical functioning (41), gait (42), balance (42), quality of life (41, 43), cognition (44), and non-motor symptoms such as depression (43). Thus, our results are consistent with the hypothesis that reduction of physical activity, through restriction of both social life and physical exercise, favored negative health consequences in people living with PD (45), such as the occurrence of major clinical outcomes and possibly an increase in the risk of death.

We did not observe any impact on the AP and VP cohorts, but a statistically not significant trend of higher risk for infections and heart failure was found. This could be explained by a limited statistical power. Moreover, as people with AP have usually a more rapid disease progression than those with PD (46) and possibly are more often bedridden, mobility restrictions could have had a limited impact on the risk for injuries in this cohort.

The main strength of our study is the clinical diagnosis performed by neurologists during the day-by-day clinical activity. In a retrospective study, diagnostic assessment by a qualified neurologist is highly preferable than relying on other methods based on administrative claims data only. Our study has some limitations, too. The diagnostic criteria applied were not the most recent published criteria for PD and progressive supranuclear palsy. This fact might have led to the misdiagnosis between the categories of PD and AP, which, however, presumably occurred in few people. Moreover, the ParkLink cohort did not include the whole population of PD or parkinsonism in the area, since not all neurologists are participating in the study. However, including unselected patients with PD or parkinsonism covering both public and private sectors, as well as community and hospital-based services, we think that our sample is representative of the entire spectrum of the disease at the population level. Finally, data on some potential covariates/confounders, such as socioeconomic status and caregiver situation, were not available, and the reduction of healthcare services was defined by a non-validated measure.

The COVID-19 pandemic is an unprecedented “natural experiment” providing the opportunity to learn more about draconian national governmental decisions and healthcare system rearrangement (4) during public health emergencies. In 2020, ~1 million excess deaths occurred in high-income countries. This outcome reflects, together with mechanisms of the direct effects of the pandemic, also the indirect effects due to associated policy measures (47). Some speculated that subjects with chronic and severe neurodegenerative diseases could have been most seriously impacted by the indirect consequences of the epidemic (5). We observed a clear chronological association between mobility restrictions, regulatory closure of services (particularly regarding physiotherapy), and major clinical outcomes in a cohort of highly vulnerable patients, such as people with PD, that was not observed in a control population. Such findings may be considered by decision-makers during possible future emergencies (such as epidemics and extreme climate changes) when planning substantial health care and social rearrangements applied to large populations. Patients with chronic conditions, such as PD, should be considered vulnerable subjects and safeguarded as such, and strategies to maintain their physical activity should be implemented.

## Data Availability Statement

The datasets presented in this study can be found in online repositories. The names of the repository and accession number can be found at: Zenodo, https://doi.org/10.5281/zenodo.6023524.

## Ethics Statement

The studies involving human participants were reviewed and approved by the Ethics Committee of the LHTB on June 25, 2015 (Reference Code: 15401). The patients/participants provided their written informed consent to participate in this study.

## ParkLink Bologna Collaborators

Emanuela Azzoni (AUSL di Bologna, Bologna, Italy), Francesca Baschieri (IRCCS Istituto delle Scienze Neurologiche di Bologna, Bologna, Italy), Marzio Bellan (Bologna, Italy), Lidia Bettelli (AUSL di Bologna, Bologna, Italy), Giuseppe Bonavina (Montecatone Rehabilitation Institute, Imola, BO, Italy), Sabina Capellari (IRCCS Istituto delle Scienze Neurologiche di Bologna, Bologna, Italy), Sabina Cevoli (IRCCS Istituto delle Scienze Neurologiche di Bologna, Bologna, Italy), Piero de Carolis (Bologna, Italy), Danilo Di Diodoro (Bologna, Italy), Giovanni Fabbri (AUSL di Bologna, Bologna, Italy), Renata Ferrara (Bologna, Italy), Anna Sandra Gabellini (Bologna, Italy), Pietro Guaraldi (IRCCS Istituto delle Scienze Neurologiche di Bologna, Bologna, Italy), Fabiola Lucchi (AUSL di Bologna, Bologna, Italy), Barbara Mostacci (IRCCS Istituto delle Scienze Neurologiche di Bologna, Bologna, Italy), Roberta Pantieri (IRCCS Istituto delle Scienze Neurologiche di Bologna, Bologna, Italy), Gaetano Procaccianti (Bologna, Italy), Rita Rinaldi (IRCCS Istituto delle Scienze Neurologiche di Bologna, Bologna, Italy), Giovanni Rizzo (IRCCS Istituto delle Scienze Neurologiche di Bologna, Bologna, Italy), Tommaso Sacquegna (Bologna, Italy), Giuseppe Samoggia (AUSL di Bologna, Bologna, Italy), Elisa Stivanello (AUSL di Bologna, Bologna, Italy), Antonella Tempestini (AUSL di Bologna, Bologna, Italy), and Carmelina Trocino (IRCCS Istituto delle Scienze Neurologiche di Bologna, Bologna, Italy).

## Author Contributions

LV, FB, LB, CZ, EB, RD'A, and FN conceptualized, designed the study, and contributed to the critical revision of the manuscript. LV, GC-B, PC, GG, MG, GL, SN, LS, CS, RD'A, and FN performed the execution of the study. FB, LB, and CZ prepared the study data and performed the statistical analysis. LV is the guarantor of the study, attests that all the listed authors meet authorship criteria, and no other meeting criteria have been omitted. All authors contributed to the interpretation of the results and approved the final manuscript.

## Dedication

This study is dedicated to the memory of Mr. Angelo Spiga, president and founder of the Associazione Parkinsoniani Pianura Est.

## Conflict of Interest

GC-B has received honoraria for speaking from AbbVie, Zambon, and Bial. The remaining authors declare that the research was conducted in the absence of any commercial or financial relationships that could be construed as a potential conflict of interest.

## Publisher's Note

All claims expressed in this article are solely those of the authors and do not necessarily represent those of their affiliated organizations, or those of the publisher, the editors and the reviewers. Any product that may be evaluated in this article, or claim that may be made by its manufacturer, is not guaranteed or endorsed by the publisher.
